# Supplementary material for: Prevalence of offering menopause hormone therapy among primary care doctors and its associated factors: A cross-sectional study
Source: PLoS One. 2024 Sep 25;19(9):e0310994. doi: 10.1371/journal.pone.0310994 (PMC11423979; doi:10.1371/journal.pone.0310994)
Supplement: S1 File — (DOCX) [file pone.0310994.s001.docx]

## S1 Questionnaire on Data Collection

Title: Prevalence of offering menopause hormone therapy among primary care doctors and its associated factors: A cross-sectional study

Section I - Sociodemographic characteristic

1. How old are you? *

1. What is your gender? *

-Male

-Female

1. How many years of practice in a primary care setting? *

1. What is your ethnicity? *

-Malay

-Chinese

-Indian

-Other:

1. What is your position? *

-Medical officer

-Family Medicine Specialist

1. Personal experience with menopause symptom* May select all that apply

-Personal menopausal symptom

-Family with menopause symptom

1. How many patients have you encountered with symptomatic menopause in a month? (estimation) *

1. Do you have menopause hormone therapy preparation in your practice? *

-Yes

-No

Section II – Preference and Practice

1. Your preferred treatment for menopause symptoms is: (select one) *

-Menopause hormone therapy (MHT)

-Non-hormonal medication (e.g., SSRI, gabapentin)

-Complementary and alternative therapy (e.g., herbal, massage, acupuncture, yoga, meditation)

-Lifestyle modification (regular exercise, adequate sleep, and a balanced diet)

1. In your actual practice, how you manage women who presented to you with menopause symptoms (vasomotor symptoms, fatigue, irritability, sleep problem, mood problem, sexual problem, or bladder problems related to menopause) in the past 12 months, considering the situation in your clinic? *

|  | Yes | No | Sometimes |
| --- | --- | --- | --- |
| Discuss with the patient regarding menopause hormone therapy |  |  |  |
| Prescribe menopause hormone therapy |  |  |  |
| Refer the patient to a tertiary centre or gynaecology clinic for menopause hormone therapy |  |  |  |
| Refer the patient for cognitive behaviour therapy |  |  |  |
| Prescribe non-hormonal medication (SSRI, gabapentin) |  |  |  |
| Discuss complementary and alternative therapy (yoga, herbal, acupuncture, massage, meditation) |  |  |  |
| Advise for lifestyle modification (regular exercise, adequate sleep, and a balanced diet) |  |  |  |

Section III: Patients’ clinical profile Section IIIA - Patient condition not to offer MHT

1. Would you offer MHT for a patient with the following condition? *

|  | Yes | No | Unsure |
| --- | --- | --- | --- |
| Personal history of breast cancer |  |  |  |
| Family history of breast cancer |  |  |  |
| Patient history of venous thromboembolism |  |  |  |
| Hypertension (Well Controlled) |  |  |  |
| Hypertension (Poorly Controlled) |  |  |  |
| Dyslipidaemia (Well Controlled) |  |  |  |
| Dyslipidaemia (Poorly Controlled) |  |  |  |
| Diabetes Mellitus (Well Controlled) |  |  |  |
| Diabetes Mellitus (Poorly Controlled) |  |  |  |
| Obesity |  |  |  |
| Patient history of atherosclerotic disease |  |  |  |
| Patient history of uterine cancer (in remission) |  |  |  |
| Patient's concern regarding breast cancer |  |  |  |

Section 3B Patient clinical condition to offer MHT

1. Would your offer MHT for which of the menopausal-related problem.

|  | Yes | No | Unsure |
| --- | --- | --- | --- |
| Vasomotor symptoms |  |  |  |
| Atrophic vaginitis |  |  |  |
| Reduced libido |  |  |  |
| Dyspareunia |  |  |  |
| Incontinence |  |  |  |
| Mood disorder (Depression/Anxiety) |  |  |  |
| Muscular skeletal pain |  |  |  |
| Treatment of osteoporosis |  |  |  |

Section 3C - Patient Age to be offered MHT

1. Would you offer MHT for the patient in the following age group?

|  | Yes | No | Unsure |
| --- | --- | --- | --- |
| <40 years old |  |  |  |
| 40-49 years old |  |  |  |
| 50-55 years old |  |  |  |
| 56-60 years old |  |  |  |
| >60 years old |  |  |  |

Section IV - Perceived barrier in MHT offering

1. The difficulties/ barriers to discussing/offering MHT that you experience are: *

|  | Yes | No |
| --- | --- | --- |
| Time constraints when discussing MHT |  |  |
| Patient concern regarding the use of MHT and breast cancer |  |  |
| Patient concerns regarding the use of MHT and other (non-breast cancer) potential risks. |  |  |
| PCDs’ concerns regarding side effects of MHT |  |  |
| MHT is not widely available |  |  |
| Lack of information regarding MHT for patient |  |  |
| No experience in prescribing MHT |  |  |
| Patient preferences for complementary/ alternative therapies |  |  |
| MHT is expensive |  |  |

Section 5 Likelihood of self-usage of MHT and recommendation to family and friends.

To what extent do you agree with this statement

1. Assume you have menopause symptoms. How likely would you take menopause hormone therapy (if you do not have any contraindications) *

-Very likely

-Likely

-Neutral

-Unlikely

-Very Unlikely

1. Would you recommend menopause hormone therapy to your family members or friends if they are symptomatic? (If they are without contraindication) *

-Very likely

-Likely

-Neutral

-Unlikely

-Very Unlikely

Section 6 – Organization support to promote menopause management

1. Are you aware of CPG's availability on "Management of menopause in Malaysia”?

-Yes

-No

1. Are you able to keep up with current recommendations/ guidelines/evidence on menopause treatment? *

-Yes

-Moderately

-No

1. Do you receive any training in menopause management for the past 12 months? (Course, CME, Webinar, Attachment) *

-None

-Once

-Twice or more

-Attachment in menopause clinic
