# Supplementary material for: Prevalence of offering menopause hormone therapy among primary care doctors and its associated factors: A cross-sectional study
Source: PLoS One. 2024 Sep 25;19(9):e0310994. doi: 10.1371/journal.pone.0310994 (PMC11423979; doi:10.1371/journal.pone.0310994)
Supplement: Attachment — Submitted filename: Response to Reviewers-20.8.2024.docx [file pone.0310994.s002.docx]

Answers to editors and reviewers

Journal Requirements:

1. When submitting your revision, we need you to address these additional requirements.

Please ensure that your manuscript meets PLOS ONE's style requirements, including those for file naming. The PLOS ONE style templates can be found at

[https://journals.plos.org/plosone/s/file?id=wjVg/PLOSOne_formatting_sample_main_body.pdf](about:blank) and 
[https://journals.plos.org/plosone/s/file?id=ba62/PLOSOne_formatting_sample_title_authors_affiliations.pdf](about:blank)

***We have checked the requirements and followed the templates given.***

2. We note that you have indicated that there are restrictions to data sharing for this study. For studies involving human research participant data or other sensitive data, we encourage authors to share de-identified or anonymized data. However, when data cannot be publicly shared for ethical reasons, we allow authors to make their data sets available upon request. For information on unacceptable data access restrictions, please see [http://journals.plos.org/plosone/s/data-availability#loc-unacceptable-data-access-restrictions](about:blank#loc-unacceptable-data-access-restrictions).

Before we proceed with your manuscript, please address the following prompts:

a) If there are ethical or legal restrictions on sharing a de-identified data set, please explain them in detail (e.g., data contain potentially identifying or sensitive patient information, data are owned by a third-party organization, etc.) and who has imposed them (e.g., a Research Ethics Committee or Institutional Review Board, etc.). Please also provide contact information for a data access committee, ethics committee, or other institutional body to which data requests may be sent.

b) If there are no restrictions, please upload the minimal anonymized data set necessary to replicate your study findings to a stable, public repository and provide us with the relevant URLs, DOIs, or accession numbers. Please see [http://www.bmj.com/content/340/bmj.c181.long](about:blank) for guidelines on how to de-identify and prepare clinical data for publication. For a list of recommended repositories, please see [https://journals.plos.org/plosone/s/recommended-repositories](about:blank). You also have the option of uploading the data as Supporting Information files, but we would recommend depositing data directly to a data repository if possible.

Please update your Data Availability statement in the submission form accordingly.

***Revision on Data availability statement as follows:***

***The data contains potentially identifiable information about healthcare providers, including age, gender, position, place of practice, years of service, and menopause experience within the study population. Contact information for a data access: Medical Research and Ethics Committee, National Institutes of Health, Ministry of Health Malaysia, Block A, Level 2, No 1, Jalan Setia Murni U13/52, Seksyen U13, Setia Alam, 40170, Shah Alam, Selangor, Malaysia Telephone: +603-3362 8398.*** [***mrecsec@moh.gov.my***](about:blank)***.***

3. Please include your full ethics statement in the ‘Methods’ section of your manuscript file. In your statement, please include the full name of the IRB or ethics committee who approved or waived your study, as well as whether or not you obtained informed written or verbal consent. If consent was waived for your study, please include this information in your statement as well.

**Revision done on page 5 line 7 and 8 on ethical approval.**

***Ethical approval for this study was obtained from the Medical Research and Ethics Committee (MREC), Ministry of Health Malaysia (NMRR ID-22-00581-UD6).***

**Revision done on page 6 line 11-12 on the consent.**

***The first part of the questionnaire consisted of the information about the study and consent. Respondent required to check “Agree” on the consent to proceed with the online survey.***

4. Please review your reference list to ensure that it is complete and correct. If you have cited papers that have been retracted, please include the rationale for doing so in the manuscript text or remove these references and replace them with relevant current references. Any changes to the reference list should be mentioned in the rebuttal letter that accompanies your revised manuscript. If you need to cite a retracted article, indicate the article’s retracted status in the References list and also include a citation and full reference for the retraction notice.

***We have reviewed the reference list and there is no retracted paper been cited.***

**Reviewers’ comments**

| Reviewers’ comments | Answers to reviewers |
| --- | --- |
|  |  |
| **Reviewer #1:** Well done to the authors in doing this important study.  In the method, suggest to include brief input of the outcome on the content and face validity of the questionnaire if there were any issues?  Please standardize the term use ie the doctors in-charge or the in-charge doctors?  p value less than (to use symbol <) | Thank you to the reviewer for acknowledge this study.  We have added more details for clarity. (Page 6, line 8 to line 10)  *Feedback from the pilot study led to minor corrections, primarily in grammar and wording, to enhance clarity and ensure the questions were clearly understood by the respondents.*  Thank you for the suggestion. We have standardised the term using ‘doctors in charge’ throughout the paper.  Thank you. Revision done as suggested. (Page 7, line 5)  *The factors with p value <0.25 in simple logistic regression analysis were included in the multivariate model. [12]* |
| **Reviewer #2:** Overall it's a very good study and very relevant to primary care. As a pioneer study, i think subsequent study can explore the reason for low prescription of MHT in relation to patients perception towards menopause symptoms and MHT.  I just want clarification regarding the questionnaire used. in the manuscript its written adapted from previous study and given the reference number (11). But in the references, reference number 11 is about 'multiple logistic regression. ( 11. Jr D, Lemeshow S, Sturdivant R. The Multiple Logistic Regression Model. In 2013. p. 35–47. ) I can't find the reference to the questionnaire used in this study.  And what step they do... is it only content validation? what language the original questionnaire used. Please provide me the original one and the method you use to validate the questionnaire. | Thank you for the suggestion. We would consider this in the subsequent study.  Thank you for informing this error. We have rechecked the references and correction done accordingly.  The revised reference for the questionnaire is still number 11 (Yeganeh L, Boyle J, Teede H, Vincent A. Knowledge and attitudes of health professionals regarding menopausal hormone therapies. Climacteric. 2017 Aug;20(4):348–55.)  The earlier reference number 11, and subsequent reference has been revised accordingly (page 25 to 27)  We have added more details for clarity. regarding the process in validating this questionnaire. We did not proceed with exploratory/confirmatory factor analysis or reliability testing as the questionnaire items were analyzed individually as categorical data, and no composite scoring was used. (Page 6, line 1 to line 10)  *The tool used in this study is a questionnaire originally developed by previous researchers to assess health professionals' attitudes regarding menopause hormone therapy in Australia.[11] The questionnaire is in English and permission was obtained from the original authors to use it in our study. To better suit our local context,* *we made some adaptations to the original questionnaire. The questionnaire items were analyzed individually as categorical data, and no composite scoring was used. Content validation of the adapted questionnaire was performed by three senior family medicine specialists. Additionally, a pilot study was conducted involving 30 health professionals, who were excluded from the final sample. Feedback from the pilot study led to minor corrections, primarily in grammar and wording, to enhance clarity and ensure the questions were clearly understood by the respondents.*  For the original questionnaire, we obtained the permission from the author to be use in the study, however we did not have permission to distribute it to other party.  The original article can be found in DOI: [10.1080/13697137.2017.1304906](about:blank)  The questionnaire items were analysed individually as categorical data, and no composite scoring was used. |
